# T-Cell redirecting bispecific antibodies: a review of a novel class of immuno-oncology for advanced prostate cancer

**DOI:** 10.1080/15384047.2024.2356820

**Published:** 2024-05-27

**Authors:** Julia Palecki, Amman Bhasin, Andrew Bernstein, Patrick J. Mille, William J. Tester, Wm. Kevin Kelly, Kevin K. Zarrabi

**Affiliations:** a Department of Internal Medicine, Thomas Jefferson University Hospital, Philadelphia, PA, USA; b Department of Medical Oncology, Sidney Kimmel Cancer Center, Thomas Jefferson University Hospital, Philadelphia, PA, USA

**Keywords:** Prostate cancer, immunotherapy, Bispecific antibody, PSMA, STEAP1

## Abstract

Novel T-cell immunotherapies such as bispecific T-cell engagers (BiTEs) are emerging as promising therapeutic strategies for prostate cancer. BiTEs are engineered bispecific antibodies containing two distinct binding domains that allow for concurrent binding to tumor-associated antigens (TAAs) as well as immune effector cells, thus promoting an immune response against cancer cells. Prostate cancer is rich in tumor associated antigens such as, but not limited to, PSMA, PSCA, hK2, and STEAP1 and there is strong biologic rationale for employment of T-cell redirecting BiTEs within the prostate cancer disease space. Early generation BiTE constructs employed in clinical study have demonstrated meaningful antitumor activity, but challenges related to drug delivery, immunogenicity, and treatment-associated adverse effects limited their success. The ongoing development of novel BiTE constructs continues to address these barriers and to yield promising results in terms of efficacy and safety. This review will highlight some of most recent developments of BiTE therapies for patients with advanced prostate cancer and the evolving data surrounding BiTE constructs undergoing clinical evaluation.

## Introduction

Prostate cancer (PCa) is the second most common cause of cancer-related death for men in the United States.^1^ An estimated 288,300 new cases of PCa leading to 34,700 deaths occurred in 2023 in the United States.^2^ Localized PCa is often treated effectively with surgery and/or radiotherapy.^3^ Active surveillance can be considered in low-risk or select intermediate-risk patients.^4,5^ Androgen ablation therapy remains the backbone of therapy for metastatic disease, though the development of castrate resistance is an inevitable reality for most patients.^6^ The incidence of metastatic castrate-resistant prostate cancer (mCRPC) has increased in recent years and the disease remains incurable, despite ongoing advancements in treatment strategies improving survival.^7^ Androgen receptor signaling inhibitors and chemotherapeutic options such as taxanes have proven efficacy and can palliate disease as does radiotherapy with radium-233, though the long-term benefit is limited, and treatment-associated toxicity is abundant.^8–10^ More recently, PSMA-targeted radioligand therapies such as^177^Lu-PSMA-617 have demonstrated promising results and highlight the effective strategy of tailoring therapy to a selective tumor associated antigen.^11^

Immunotherapies such as immune checkpoint inhibitors, which have revolutionized the treatment of certain solid tumors and hematologic malignancies, have demonstrated limited efficacy in patients with PCa.^12,13^ In recent years, T-cell engager immunotherapies including chimeric antigen receptor (CAR) T-cell therapies and bispecific T-cell engager (BiTE) therapies have emerged as promising modalities for the treatment a variety of hematologic malignancies, and hold promise of demonstrating efficacy in patients with PCa.^14,15^ In this review, we highlight novel BiTE therapies under development for patients with PCa, focusing on early reports of their efficacy and tolerability. We aim to share our perspective on BiTE successes and pitfalls, as well as to reflect on how BiTE therapies will shape the future of the treatment of mCRPC.

## T-cell redirection as an immunotherapeutic strategy

T-cell engager therapies are a class of immunotherapies that enhance the ability of the patient’s native immune system to recognize and target cancer cells. Currently, the only FDA-approved immunotherapy for the treatment of advanced PCa is Sipuleucel-T, an autologous cellular immunotherapy thought to work via antigen-presenting cell-induced stimulation of the T-cell immune response against prostatic acid phosphatase, an antigen expressed in PCa.^16–18^ The success with Sipuleucel-T has been limited thus far and overall clinical benefit in the current era of novel therapies has not be well defined. Despite its limitations, Sipuleucel-T serves as a proof of principle that T-cell redirecting therapy may be effective and immunotherapy has the potential for transformative impact in PCa.

More contemporary T-cell redirection strategies include CAR T-cell therapy and BiTEs. CAR-T entails engineering a patient’s autologous T-cells to express a chimeric antigen receptor, which enables them to recognize and bind to specific antigens on the surface of cancer cells. CAR T-cell therapy has demonstrated tremendous success in the treatment of certain hematologic malignancies and is now being studied in solid tumors, including PCa, with limited progress due to numerous challenges including complexities inherit to the immune microenvironment of various solid tumors.^19–23^ Early studies of CAR-T in PCa include multiple ongoing phase I studies including a PSCA-targeted 4-1BB-co-stimulated CAR T-cell therapy (NCT05805371) and multiple PSMA-targeting CAR T-cell therapies (NCT03089203, NCT04249947).^24,25^

Like CAR T-cell therapy, BiTE immunotherapy also redirects T-cells against selective TAAs and has shown success in hematologic malignancies, thus opening the door for their study in solid tumors such as PCa.^26,27^ Bispecific antibodies are engineered to harbor two distinct scFv binding domains capable of simultaneously recognizing and binding distinct antigens on two different cell types. In the context of PCa, bispecific antibodies can be engineered to target both antigens expressed on PCa cells as well as various T cell receptors including CD3 and CD28. BiTEs serve as a bridge connecting prostate tumors to T-cells, thus directly stimulating cytotoxic T-cell activity without relying on the interaction between the T-cell receptor with major histocompatibility complex co-stimulation. Subsequent T-cell activation triggers a cytotoxic immune response targeted specifically against cells that express the target antigen.^28–30^

There are two structural formats to bispecific antibodies undergoing development in PCa; (1) single-chain variable fragment-based antibodies, and (2) full-length IgG-based antibodies. Single-chain variable fragments are generated by fusing the variable domains of the IgG heavy chain and light chains of anti-tumor and anti-immune cell antibodies through polypeptide linkage while retaining their binding ability. IgG-like full-size bispecific antibodies are produced through heterodimerization of complete heavy chain and light chains from anti-tumor and anti-immune cell antibodies, retaining a structure similar to native antibodies.^26,31^

PCa is characterized by an immunologically “cold” tumor microenvironment (TME) which hinders the immune response, allowing for immune evasion and disease progression. The TME of PCa is characterized by diminished levels of tumor-infiltrating lymphocytes, decreased activation of antigen-presenting cells, and increased presence of immunosuppressive cell types, including myeloid-derived suppressor cells. Additionally, PCa exhibits a low tumor mutational burden, thereby limiting the availability of neoantigens for immune recognition.^32–34^ Because of this immunologically “cold” TME, immune checkpoint inhibitors, which have exhibited remarkable efficacy in other solid tumors, have yielded disappointing outcomes in PCa. The notable exception is pembrolizumab, an IgG4 kappa monoclonal antibody that inhibits the programmed death-1 (PD-1) receptor, which has received FDA approval for select patients with mCRPC characterized by high tumor mutational burden, high microsatellite instability, or deficient mismatch repair.^35–37^ By simultaneously binding immune effector cells with TAAs to generate antitumor response, T-cell redirected bispecific antibodies are optimally designed to overcome the “cold” TME of PCa, and thus enable the successful use of immunotherapy in PCa.

## Prostate tumor associated antigens as epitopes for BiTes

A TAA is a molecular structure, typically a protein, that is overexpressed by malignant cells that can be recognized by the immune system as a target against which to mount an immune response.^38^ The success of immunotherapy in the treatment of PCa hinges on the identification and effective targeting of optimal TAAs to generate a robust and specific anti-tumor response.^39^ The ideal TAA exhibits the following features: it is readily accessible on the surface of tumor cells, ensuring detection by immune effector cells; it is prevalent across all malignant cells within a given tumor, reducing the risk of immune escape; and it exhibits conservation across patients with the same cancer type, enabling the development of broadly applicable immunotherapies. Furthermore, the ideal TAA demonstrates tumor specificity with exclusive, or at least predominant, expression on tumor cells rather than on non-cancerous tissue.^40,41^ The development of immunotherapies against TAAs with high tumor specificity is essential for minimizing off-tumor activation, preventing damage to healthy cells, and thus allowing for the safe and tolerable use of the drug.^42,43^ Fortunately, several promising TAAs have been identified in PCa including PSMA, PSCA, hK2 and STEAP1, among others.^38^ This positions PCa as an ideal candidate for the investigation of BiTE therapies and has spurred the development of numerous novel BiTE constructs with unique structures that target the aforementioned TAAs.

Prostate-specific antigen (PSA) is almost exclusively expressed in prostate epithelial cells and is detected in the majority of PCa tissues. While PSA is widely used as a serum marker for the diagnosis and monitoring of PCa, it has not emerged as a feasible immunotherapy target. Notably, prostate-specific membrane antigen (PSMA) stands out as a promising TAA for PCa immunotherapy. PSMA is an integral membrane glycoprotein minimally detected on non-neoplastic prostate cells but highly expressed in the majority of prostate tumors.^44^ While PSMA can be found in other nonmalignant tissue including the salivary gland, central nervous system, small intestine, breast epithelium, and renal tubular epithelium, its expression in non-prostate tissues is significantly lower.^45,46^ The significant overexpression of PSMA in tumor cells as well as the extracellular location that facilities recognition by immune cells makes this TAA an optimal target, and several PSMA targeting BiTEs have been developed and implemented in early phase clinical trials.

Prostate Stem Cell Antigen (PSCA) is a glycosylphosphatidylinositol-anchored cell surface glycoprotein that is expressed in basal and secretory epithelial cells of the prostate. PSCA expression is detectable in >80% of primary malignant prostate samples and bone metastases. PSCA expression is increased in prostate tumors compared with corresponding nonmalignant prostatic tissue, and expression correlated with higher Gleason scores.^47,48^ Upregulation of PSCA was also noted in non-organ confined tumors and seminal vesicle invasion as compared to tumors restricted to the prostate. Six-transmembrane epithelial antigen of the prostate 1 (STEAP 1) is a transmembrane protein predominantly expressed in the prostate epithelium but has been detected in the colon and liver at lower levels of expression. STEAP 1 is overexpressed in different stages of PCa as well as other tumors including bladder, colon, and ovarian cancer.^45,49^ Human kallikrein 2 (hK2) is a serine protease encoded by the KLK2 gene that is expressed in both malignant and nonmalignant prostate tissue. However, hK2 is overexpressed in PCa cells and its levels correlate with the severity of the disease, establishing it as a specific and dependable target for potential immunotherapeutic interventions.^50^

## Early generation BiTEs in prostate cancer

AMG 212 (pasotuxizumab) is an anti-PSMA/CD3 BiTE and was one of the early BiTE immunotherapies employed in PCa. In a preclinical study, AMG212 was able to effectively bind to PSMA-expressing cells as well as human T-cells to trigger T-cell activation, cytokine release, and antigen-dependent target cell lysis. Moreover, AMG212 effectively delayed tumor growth and led to tumor shrinkage in human PCa xenograft models.^51^ A phase I trial (NCT01723475) enrolled 47 patients with mCRPC that received AMG212, either as a subcutaneous (SC) or continuous intravenous (cIV) formulation (SC *n* = 31, IV *n* = 16). All enrolled patients had treatment failure after ≥1 prior taxane regimen. The most common treatment-emergent adverse events (TRAE) reported were fever, injection site reaction, chills, and fatigue. TRAE of grade ≥3 occurred in 87% (27/31) of patients in the SC cohort, with the most common being anemia (39%) and decreased lymphocyte count (26%). TRAE of grade ≥3 occurred in 81% (13/16) of patients in the cIV cohort, with the most common being decreased lymphocyte count (44%) and infection (31%). All 30 of 30 patients who received ≥1 dose of SC AMG212 developed anti-drug antibodies (ADAs) with a median onset of 22 days after treatment. The ADAs were sustained and not responsive to mitigating measures such as glucocorticoid treatment. Due to the high rate of ADAs, further evaluation of the SC route of administration was discontinued. No ADAs were detected in the cIV cohort.^52,53^ It was determined that the high-titer, sustained ADA response in the SC arm was due to the immunogenic route of administration and not the T-cell epitopes within the AMG212 amino acid sequence.^54^ In terms of efficacy, the median best overall PSA response in the SC cohort was a decline of 25% with a third of patients showing an initial >50% decline in PSA values, though PSA responses were not sustained and typically rose to higher levels than baseline levels over time, possibly due to development of neutralizing ADAs. In the cIV cohort, there was a dose-dependent decline in serum PSA, and 14 patients showed a decline in PSA during treatment. A > 50% decline was seen in 3/9 of patients at higher doses (in the 20-, 40- and 80-μg cohorts). Two patients had long-term responses with sustained >50% reductions in PSA for 50 weeks and >80 weeks.^52,53^ AMG212 was the first BiTE monotherapy in clinical study to demonstrate efficacy in patients with PCa. Ultimately AMG212 was discontinued due to the high prevalence and severity of side effects, and further study of AMG212 was halted in favor of novel BiTE constructs.

AMG160 (acapatamab) is a second-generation anti-PSMA/CD3 BiTE featuring an additional Fc fragment fused to the antibody core intended to prolong drug half-life thus allowing for a more practical delivery through biweekly IV infusions.^55–57^ In a phase I study (NCT03792841) study of patients with mCRPC refractory to prior novel hormonal therapy and taxane treatment, 32 patients received short IV biweekly infusions of AMG 160. Cytokine release syndrome (CRS) was the most common adverse effect (27 patients) and presented with associated fever, transaminitis, and hypotension. CRS symptoms occurred primarily during cycles 1–2, and were managed with standard mitigation approaches. RECIST (Response Evaluation Criteria In Solid Tumors) responses among 18 patients with measurable disease included 1 confirmed partial response, 5 patients with stable disease, and 5 patients with disease progression. PSA reductions occurred in 15/24 (63%) of evaluable patients. In the two highest dose-level groups, PSA reductions >50% occurred in 6/10 (60%) of patients.^58^ Clinical study with AMG160 was ultimately suspended in favor of AMG340, and next-generation an anti-PSMA/CD3 BiTE with a low-affinity anti-CD3 arm aimed at reducing immune activation and CRS.^59^ Unfortunately, AMG340 was recently discontinued for unspecified reasons and the ongoing phase 1 dose-escalation study in mCRPC (NCT04740034) is suspended indefinitely.

APVO414 (MOR209/ES414) is an anti-PSMA/CD3 BiTE developed using ADAPTIR technology that incorporates two single-chain variable fragment homodimers, each capable of binding both CD3 and PSMA. In preclinical trials, the homodimer structures demonstrated improved half-life, stability, and potency.^60^ In a phase I study (NCT02262910), APVO414 demonstrated significant immunogenicity and the majority of patients developed neutralizing ADA’s. In the initial cohort of the dose escalation study, 7/12 (58%) patients developed ADA’s with very high titers (as high as 1:250,00). Though none of the patients had adverse reactions due to the ADA’s, patients with high ADA titers cleared the drug to undetectable levels. Regimen modification from weekly IV dosing to continuous IV infusion resulted in a slight decrease in development of ADA from 58% to 50% but dramatically decreased titers from 1:125,000 to 1:160–1:320.^61^ Ultimately, given the significant immunogenicity and lack of sufficient therapeutic benefit with APVO414 the study was discontinued.

Similarly, JNJ-63898081(JNJ-081), an anti-PSMA/CD3 BiTE developed using the innovative DuoBody platform, encountered significant issues ultimately leading to early trial closure. A phase 1 dose escalation study (NCT03926013) evaluated JNJ-081 in 39 patients with mCRPC who progressed after novel androgen targeting therapy or prior chemotherapy. JNJ-081 was initially administered by IV followed by a subsequent cohort employing a SC route. The most common TRAEs were CRS (65%), fatigue (49%), and nausea (43%). Grade 2 CRS was observed at higher doses and was partially mitigated by SC and step-up dosing. Grade 2 CRS was seen in 0/7 of patients who received doses ≤1 µg/kg weekly IV and in 60% (3/5) of patients who received a dose of 3 µg/kg weekly IV. Grade 2 CRS was seen in 75% (3/4) patients who received 30 µg/kg weekly subcutaneous without priming, but only in 25% (1/4) of patients who received higher doses with priming (i.e. 5 and 20, then 60 µg/kg). Transient PSA decreases were observed in the SC cohort at treatment doses greater than 30 µg/kg. No radiographic responses were observed. In terms of immunogenicity, ADA antibodies were detected in 2/12 patients treated by IV administration and 14/24 patients with SC administration, resulting in loss of exposure in some SC patients.^62,63^

HPN424 is a first-in-class tri-specific T-cell engager with a conventional anti-PSMA/CD3 backbone fused with a third albumin-binding Fc domain to enhance drug stability and extend serum half-life. A Phase I/IIa study (NCT03577028) evaluated HPN424 in 80 patients with mCRPC who have received >2 prior systemic therapies. The most common >3 TEAEs were AST increase (18%), ALT increase (11%), and anemia (11%). All-grade CRS occurred in 63% of patients, and there was no incidence of Grade 4 or 5 CRS. On correlative study, reduction in circulating tumor cells was seen in 32 of 56 patients (57%) with measurable CTC at baseline. Thirteen of 63 patients (21%) had PSA declines from baseline including 3 PSA50 and 2 PSA30 responses. Due to the unfavorable balance between efficacy and toxicity, further investigation of this drug was stopped^64^ (Table 1).

**Table 1.** Trials of bispecific t-cell engagers in prostate cancer with reported results.

National Clinical Trial	Phase	Drug	Intervention	Indication	Enrollment	Primary Endpoint
NCT01723475	I	Pasotuxizumab (AMG212/BAY2010 112)	PSMA x CD3	mCRPC	47	AE profile, DLT, MTD,
NCT03792841	I	Acapatamab (AMG160)	PSMA x CD3	mCRPC	212	AE profile, DLT
NCT04631601	I/II	Acapatamab (AMG160)	PSMA x CD3	mCRPC	65	AE profile, DLT
NCT04740034	I	AMG 340	PSMA x CD3	mCRPC	100	AE profile, DLT, ORR, OS
NCT02262910	I	APVO414 (MOR209/ES414)	PSMA x CD3	mCRPC	401	AE profile, MTD
NCT03926013	I	JNJ-63898081(JNJ- 081)	PSMA x CD3	mCRPC	40	AE profile, DLT
NCT03577028	I/IIa	HPN424	PSMA x CD3	mCRPC	110	DLT, ORR
NCT03927573	I	GEM3PSCA	PSCA x CD3	PCa, NSCLC, Renal Cancer, Transitional Cell Cancer	23	AE profile, DLT, MTD
NCT04077021	I	CCW702	PSMA x CD3	mCRPC	22	AE profile, DLT. ORR

AE: Adverse Effect, DLT: Dose Limiting Toxicity, mCRPC: Metastatic Castrate-Resistant Prostate Cancer, MTD: Maximum Tolerable Dose, NSCLC: Non-Small Cell Lung Cancer, ORR: Objective Response Rate, OS: Overall Survival, PCa: Prostate Cancer, PSMA: Prostate-Specific Membrane Antigen, PSCA: Prostate Stem Cell Antigen.

## Contemporary BiTEs in clinical study

AMG509, or Xaluritamig is a bispecific antibody with two identical humanized anti-STEAP1 domains as well as a single chain variable fragment anti-CD3 chain. AMG509 harbors an additional Fc domain to extend serum half-life. The targeted TAA STEAP1 is a six-transmembrane epithelial antigen that is overexpressed in approximately 80% of metastatic prostate cancers and has an independent association with poor prognosis. Importantly, STEAP1 has low levels of expression in non-neoplastic tissues, making it an ideal target for PCa therapy.^49,65–67^ Preclinical studies demonstrated AMG509-induced T-cell mediated lysis of STEAP1 expressing cancer cells in various xenograft tumor models.^68^ Results from an ongoing phase I trial of AMG509 (NCT04221542) reporting data from 97 subjects with mCRPC are highly encouraging. Over half (53%) of the patients studied had radiologically visible visceral metastases at initiation of therapy and 79% of the patients had received 3 or more prior lines of therapy, including 85% of the patients who had received prior taxane-based systemic chemotherapy. Subjects received dosing ranging from 0.001 to 2 mg as weekly or biweekly injections as part of the dose escalation. Subjects were divided into either a low-dose cohort with target doses of 0.001 mg to 0.3 mg (*n* = 45) or a high-dose cohort with target doses of 1.0–2.0 mg (*n* = 52) based on the minimal efficacy doses found in pre-clinical studies. The maximum tolerated priming dose was 0.1 mg, and the MTD of AMG 509 weekly dosing was 1.5 mg.

Responses were seen across all dose levels, with greater response rates seen at higher levels of the drug. Of the 97 subjects who received at least one dose of Xaluritamig, 49% of these patients achieved a PSA50 response. Additionally, 24% of these subjects had an objective response rate (ORR) per RECIST criteria, which was evaluable in 67 of 97 patients. These responses improved to a 59% PSA50 response rate and 41% ORR in the 52 pts in the high dose (0.75 mg or greater) cohort. Of the patients receiving high-dose therapy, 16 (36%) achieved a PSA90 response, and 13 (25%) continued on treatment for >6 months. The most common adverse effect was CRS (72%), followed by fatigue (45%), myalgia (34%), and fever (32%). The majority of patients who experienced CRS had grade 1 or grade 2 toxicity, with only two grade 3 CRS events and no grade 4 or grade 5 CRS. Twenty-six subjects (27%) in the trial received tocilizumab as part of CRS treatment. Eighteen patients (19%) discontinued the drug due toxicity, 46 patients (47%) required interruptions and reductions in dosing. There were no fatal adverse effects. One patient passed away due to a subdural hematoma secondary trauma that was deemed unrelated to treatment. Additionally, 54% of the subjects developed anti-drug antibodies (ADA), with a median onset after 3 cycles. However, the proportion of patients achieving PSA50 in the ADA-positive group was equivalent to the ADA-negative group, and as such the development of ADA was not associated with any effects blunting drug activity. The reason that ADA formation did not affect drug activity is because responses typically occurred within 4 to 8 weeks.^69–71^

These initial efficacy findings far outpace those of prior T-cell engagers in advanced PCa, which were found to have PSA50 responses ranging from 5% (JNJ-63898081 phase I trial) to 34% in the half-life extended AMG160.^62,72^ Given the significant improvement in initial efficacy data of AMG509 without significant changes in adverse effect profile or risk, this ongoing clinical trial establishes STEAP-1 as a promising immunotherapeutic TAA and AMG509 as an active agent in men with mCRPC. More mature data is highly anticipated.

REGN5678 is a first-in-class anti-PSMA/CD28 BiTE. Preliminary results from a phase I/II study (NCT03972657) examining REGN5678 in combination with anti-PD-1 antibody cemiplimab provide the first evidence of clinical activity of with use of a CD28 co-stimulatory domain as part of a bispecific antibody in solid tumors. The study enrolled 35 patients with mCRPC who had received ≥ 2 lines of systemic therapy and treated weekly REGN5678 as monotherapy for 3 weeks, followed by combination with cemiplimab until progression or toxicity. Fifty-four percent of patients had ≥ grade 3 TRAE. CRS was limited to grade 1 severity, and occurred in only six patients. Interestingly, four patients (11%) experienced a ≥ grade 3 immune-mediated adverse event and they all benefited from a PSA decline, suggesting a possible correlation. Unfortunately, two patients experienced toxicities resulting in death: 1 from acute kidney injury (not considered treatment-related) and 1 from hemophagocytic lymphohistiocytosis (considered treatment-related). Clinical efficacy was associated with increasing dose. There was minimal efficacy at lower doses with only 1/16 patients having PSA decline at treatment doses between 0.1 and 10 mg. At 30 mg, 1/4 of patients had PSA90 decline; at 100 mg, 3/8 of patients had PSA declines (of 22%, 44%, and >99%); at 300 mg, 3/4 patients had PSA decline (of 82%, 99%, and >99%). This study is ongoing and randomized phase II dosing is yet to be determined.^73^

CC1 is an anti-PSMA/CD3 BiTE featuring a distinctive IgG scaffold PSMA antibody. The CC1 antibody construct harbors enhanced dual-targeting abilities by facilitating the immune cells to infiltrate the tumor more effectively and by engaging antigens expressed not just on tumor cells but also on tumor vessels.^74^ The initial findings from an ongoing phase I trial (NCT04104607) enrolling 14 patients with mCRPC are encouraging. The most common toxicity was CRS which occurred in 79% of the patients. The CRS did not exceed grade 2 and resolved in most cases without need for tocilizumab. A rapid and profound decline of PSA levels was observed in all the patients, with up to 60% reduction compared to baseline. Three patients in the dose escalation phase received multiple treatment cycles at the highest dose level and benefited from the rapid and profound decline of elevated PSA.^75^ Altogether, CC-1 has a favorable toxicity profile and promising clinical activity.

These encouraging results have led to the initiation of an additional phase I trial (NCT05646550) employing the same CC-1 BiTE in patients experiencing biochemical recurrence of PCa.^76^ Interestingly, investigators noted a considerable increase in platelet activation associated with CC-1 treatment that was coupled with a decline in total platelet count, which they hypothesized occurred through a TGFβ-dependent process. In patients experiencing this phenomenon, there was a notable reduction in T-cell reactivity and the ability to lyse target cells. The authors speculate that simultaneously blocking the TGFβ axis to restore platelet inhibition could significantly enhance the effectiveness of CC-1 BiTE treatment.^77^

LAVA-1207 is a BiTE that binds PSMA and the Vδ2 chain of Vγ9 Vδ2-T cells, which are highly potent immune effector cells. While the development of BiTEs has largely focused on targeting standard CD3+ T cells, this novel construct utilizes γδ-T cells as an alternative target effector cell. γδ-T cells induce rapid innate-like immune responses whereas conventional effector T cells harbor more of an ability to form memory cells. γδT cell expansion have been associated with longer survival. An ongoing phase I/II clinical trial (NCT05369000) employing LAVA-1207, administered through biweekly infusions, is being conducted and is treating 16 patients with refractory metastatic castration-resistant disease. This trial has already successfully determined a maximum tolerated dose of 40 µg. Overall, LAVA-1207 demonstrated a favorable safety profile and adverse events associated with treatment were mild to moderate in severity, dose-independent, and did not lead to therapy discontinuation. Most common toxicities were fatigue, nausea, transaminitis, and infusion reactions. Preliminary data at the 8-week evaluation point reveal that of the eight patients evaluated, three exhibited stable disease.^78^ Further clinical activity data is needed, but topline results are encouraging for LAVA-1207.

HER2 BATs are anti-CD3 × anti-Her2 bi-armed activated T-cells that target HER2 tumor antigen in a non-MHC restricted manner. In a preclinical study, HER2 BAT-associated activated T cells demonstrate anti-tumor cytotoxicity, effective intratumoral trafficking, and secretion of cytokines such as IFNγ, TNFα, and GM-CSF, upon tumor engagement. Moreover, HER2 BATs demonstrate tumor targeting in HER2 low expressing prostate cell lines.^79,80^ In a phase I study (NCT03406858), there were no dose-limiting toxicities in evaluable patients. One patient demonstrated a partial response and three patients had a significant decrease in their PSA levels. These data provided a strong rationale for further study of the agent and a subsequent phase II study evaluating HER2 BATs in combination with PD-1 inhibitor pembrolizumab in patients with mCRPC previously treated with an androgen receptor axis targeting agent and prior docetaxel chemotherapy. Six of 13 evaluable patients demonstrated a PSA decline of 25% or greater and 5 of 14 patients were progression-free at 6 months. The regimen was well tolerated, and toxicities included fevers, chills, headache, nausea, and myalgias.^81^

The investigation of BiTE therapy for PCa is not limited to prostate adenocarcinoma, but also includes neuroendocrine tumors of the prostate (NEPC). AMG 757 (Tarlatamab) is an anti-delta-like ligand 3 (DLL3)/CD3 BiTE that is currently being investigated in a phase Ib clinical trial (NCT04702737). DLL3 is highly expressed in NEPCs of the prostate, especially those with treatment-emergent transformation from adenocarcinoma to high-grade NEPC.^82–84^ AMG 757 has demonstrated safety and efficacy in an ongoing phase 1 clinical trial (NCT03319940) in small-cell lung cancer^85,86^ and there is optimism surrounding its potential role for the treatment of NEPC where options are limited.

CB307 is a novel tri-specific Humabody therapeutic targeting CD137 (4-1BB), PSMA, and human serum albumin that selectively enhances immune cell activity only in the presence of PSMA-positive cells. CD137 agonism stimulates immune cell proliferation, cytokine production, and survival. In preclinical models, CB307 augmented tumor cell killing in PSMA-expressing cells, and enhanced tumor cell cytotoxicity were observed when in combination with PD-1/PD-L1 inhibition.^87^ A phase I study (NCT04839991) is currently investigating the safety and efficacy of CB307 both as monotherapy and in combination with pembrolizumab in patients with advanced and/or metastatic PSMA-positive tumors including mCRPC.

REGN4336 is a PSMAxCD3 bispecific antibody. In preclinical models, REGN4336 demonstrated strong PSMA-dependent antitumor activity that was dose-dependent. Preclinical data suggest synergy with a combination of REGN4336 and cemiplimab in castrate-resistant prostate models. An ongoing-phase I/II study (NCT05125016) is evaluating REGN 4336 as monotherapy or in combination with cemiplimab to assess safety, tolerability, and pharmacokinetics as well as to assess preliminary anti-tumor activity in patients with metastatic castration-resistant prostate cancer^88^ (Table 2).

**Table 2.** Ongoing clinical trials with bispecific t-cell engagers in prostate cancer.

National Clinical Trial	Phase	Drug	Intervention	Indication	Enrollment	Primary Endpoint	Status	Estimated Completion Date
NCT04221542	I	AMG509	STEAP1 x CD3	mCRPC	441	AE profile, DLT	Recruiting	June 2028
NCT03972657	I/II	REGN5678 + Cempilimab	PSMA x CD28	mCRPC ccRCC	297	AE, DLT, ORR	Recruiting	July 2026
NCT04104607	I	CC1	PSMA x CD3	mCRPC	86	AE profile	Recruiting	December 2024
NCT05646550	I	CC1	PSMA x CD3	Biochemical recurrence of PCa	56	AE profile, MTD, PSA response, overall/progression free survival	Recruiting	December 2025
NCT05369000	I/IIa	LAVA-1207	PSMA x Vδ2 chain of Vγ9Vδ2-T cells	mCRPC	66	AE profile, DLT	Recruiting	March 2024
NCT03406858	II	HER2 Bi-Armed Activated T-cells	HER2 x CD3	mCRPC	15	Progression-free survival	Recruiting	Completed
NCT04702737	Ib	Tarlatamab (AMG 757)	Delta-like ligand 3 x CD3	NEPC	41	AE profile, DLT, OR	Active	August 2025
NCT04839991	I	Trispecific Humabody T-cell enhancer (CB30)7	CD137 x PSMA x human serum albumin	mCRPC	70	AE profile, DLT, MTD, progression-free survival	Recruiting	September 2024
NCT05125016	I/II	REGN4336 ± Cempilimab	PSMA x CD3	mCRPC	199	AE profile, DLT, ORR	Recruiting	August 2026
NCT05441501	I	JNJ-80038114	PSMA x CD3	mCRPC	90	AE profile, DLT	Recruiting	March 2025
NCT04898634	I	JNJ-78278343	KLK2 x CD3	mCRPC	165	AE profile, DLT, PSA RR, ORR, DOR	Recruiting	November 2025
NCT06095089	I	JNJ-87189401 + JNJ-78278343	PSMA x CD28 + KLK2 x CD3	mCRPC	110	DLT, AE profile, ADA development, ORR, PSA response rate, DOR	Recruiting	June 2027
NCT05733351	I	Vudalimab (XmAb20717) + abiraterone, enzalutamide, or abiraterone + docetaxel	PD-1 x CTLA-4	mCRPC	30	AE profile, PFS, ORR, PSA RR, DOR	Recruiting	December 2027
NCT05032040	II	Vudalimab (XmAb20717)	PD-1 x CTLA-4	Ovarian cancer, clear cell carcinoma, endometrial cancer, cervical cancer, mCRPC	150	ORR	Recruiting	May 2025
NCT05588609 (Group B)	II	Zenocutuzumab + enzalutamide or abiraterone	HER2 x HER3	mCRPC	40	PSA50 response rate, ORR, PSA30 RR, rPFS, DOR, AE profile, TTR	Recruiting	March 2026
NCT05652686	I	PT217 (PT217X1101)	huDLL3 x huCD47	SCLC, LCNEC, NEPC, GEP-NET	58	AE profile, DLT, MTD, ORR	Recruiting	January 2025
NCT03761017	I	Lorigerlimab (MGD019)	PD-1 x CTLA-4	NSCLC, mCRPC, cutaneous melanoma, CRC	162	AE profile, immunogenicity, ORR, DOR, PFS, OS, PSA RR	Active, Not Recruiting	September 2024
NCT05293496	I/Ib	Vobramitamab duocarmazine (MGC018) + Lorigerlimab	ADC against B7-H3 antigen + PD-1 x CTLA-4	mCRPC, melanoma, pancreatic carcinoma, HCC, epithelial ovarian cancer, RCC	278	AE profile, ORR, development of ADA, PFS, DOR, OS, rPFS, PSA RR,	Recruiting	March 2025
NCT05585034	I	XmAb®808 + Keytruda (Pembrolizumab)	B7-H3 x CD28 + PD-1	Head and neck SCC, melanoma, NSCLC, urothelial carcinoma, ccRCC, mCRPC,epithelial ovarian cancer, TNBC, CRC	220	AE profile, DLT, ORR, PFS, DOR	Recruiting	December 2027

ADA: Anti-Drug Antibody, ADC: Antibody–Drug Conjugate, AE: Adverse Effect, ccRCC: Clear Cell Renal Cell Carcinoma, CTLA: Cytotoxic T-Lymphocyte Antigen, DLT: Dose Limiting Toxicity, DOR: Duration of Response, GEP-NET: Gastroenteropancreatic Neuroendocrine Tumors, HER2: Human Epidermal Growth Factor Receptor 2, huCD47: Human CD47, huDLL3: Human DLL3, KLK2: Kallikrein-Related Peptidase 2, LCNEC: Large Cell Neuroendocrine Cancer, mCRPC: Metastatic Castrate-Resistant Prostate Cancer, MTD: Maximum Tolerable Dose, NEPC: Neuroendocrine Prostate Cancer, OR: Objective Response, ORR: Objective Response Rate, OS: Overall Survival, PD-1: Programmed Cell Death Protein 1, PFS: Progression-Free Survival, PSA: Prostate-Specific Antigen PSCA: Prostate Stem Cell Antigen, PSMA: Prostate-Specific Membrane Antigen, rPFS: Radiographic Progression-Free Survival, RR: Response Rate, SCLC: Small Cell Lung Cancer, STEAP1: Six-Transmembrane Epithelial Antigen of Prostate 1, TTR: Time to Response

## Perspectives on BiTEs: successes and challenges

BiTE immunotherapy for mCRPC represents an emerging treatment modality with promising therapeutic potential. Despite the potential of T-cell engaging therapy, drug formulations remain in the early stages of development and the early trials reported to date demonstrated several pitfalls and triumphs in safety and efficacy. One major barrier affecting the administration of BiTE therapy is the adverse safety profile related to immune activation effects, namely CRS, immune effector-cell-associated neurotoxicity syndrome (ICANS), and on-target-off-tumor (OTOT) toxicity.

CRS occurs as a result of uncontrolled systemic inflammatory response due to excessive release of pro-inflammatory cytokines that are secreted during T-cell activation. Prophylactic treatment with dexamethasone and step-wise dose escalation has been shown to reduce the incidence of CRS.^89,90^ Similarly, ICANS toxicity is related to excessive immune activation but with unclear pathophysiology which is hypothesized to occur secondary to local CNS inflammation. ICANS presents with a wide range of clinical manifestations ranging from headaches to altered mental status and encephalopathy.^91–93^ Finally, OTOT occurs when TAA target recognition in non-cancerous tissue results in an unintentional cross-reactivity and cell lysis. Prevention of OTOT can be avoided through selection of TAA targets with high expression and specificity for neoplastic tissues and with low levels of physiologic expression in non-neoplastic tissue.^94,95^ These treatment-related toxicities have resulted in the early termination of several clinical trials; however, newer generations BiTE constructs have shown improved safety tolerability with effective dosing of therapy.

As a class of therapy, BiTE therapies have the potential to overcome immune evasion, a significant obstacle observed in PCa treatment. Tumors have demonstrated the ability to avoid immune-mediated elimination through several mechanisms of immune evasion including loss of antigenicity, loss of immunogenicity, and creation of an immunosuppressive tumor microenvironment.^96–98^ Advanced PCa cells downregulate MHC class I molecules, resulting in the loss of antigenicity and avoidance of native immune detection.^99,100^ Furthermore, PCa cells secrete immunosuppressive cytokines and recruit regulatory T-cells, fostering an immunologically “cold” TME.^96,101,102^ BiTE therapy offers hope to overcome these immune evasion mechanisms early trials have been effective in converting the PCa TME to a “hot” environment rich in immunogenic factors. Despite varied treatment responses in early clinical trials, new generations of BiTE therapies have demonstrated improved treatment efficacy. However, similar to other classes of immunotherapy, BiTE therapies are vulnerable to treatment resistance through TAA target downregulation, leading to decreased long-term efficacy. As such, multimodal combination therapy with other immunotherapy treatment modalities, such as CAR-T and immune checkpoint inhibitors, may demonstrate synergistic effects with improved durability of response.

BiTE therapy initially faced several logistical challenges due to the short half-life of initial formulations, which required continuous IV administration. Attempts at subcutaneous administration resulted in the rapid formation of ADAs. Fortunately, new BiTE constructs have been engineered with half-life-extended (HLE) formulations that have significantly increased the half-life and allowed for intermittent administration of therapy. HLE formulations have the potential for “off-the-shelf” drug manufacturing, which will allow BiTE therapy to be widely distributed with lower production costs and no individualized drug creation necessary, unlike CAR-T.^103^

The growth of immuno-oncology and the initial efficacy of BiTE therapy has incited the creation of a variety of new drug formulations including CAR-T, simultaneous multiple interaction T-cell engagers (SMITE), dual-affinity retargeting bispecific antibodies (DART), CAR-NK, bispecific killer engagers (BiKE), and tri-specific killer engager agents (TriKE). Similar to BiTE therapy, CAR-T represents another line of T-cell redirecting therapy that has displayed robust anti-tumor activity in hematologic malignancies and has shown promising potential in the treatment of solid tumor malignancies in early clinical trials. Barriers to CAR-T therapy include personally engineered drug creation for individual patients, lymphodepletion prior to administration in immunocompromised hosts, and adverse safety effects similar to those of BiTE therapy.^104,105^ CAR-NK therapy is an extension of CAR-T therapy that alternatively utilizes activation of natural killers (NK) cells of the immune system in lieu of T-cells. This therapy has demonstrated the retained innate ability of NK cells to identify target neoplastic cells with downregulated TAAs as part of the immune evasion tactics of tumor cells. Additionally, the decreased life span of NK cells has been shown to improve the safety profile of these drugs and favorable pre-clinical studies have generated exciting potential for future clinical trials.^106,107^ SMITE and DART therapies utilize the engineering framework of BiTE therapy to target multiple TAAs simultaneously with the goal of preventing acquired tumor resistance, which has been observed to occur for BiTE therapies employed in hematologic malignancies.^103,106^ Contrastingly, BiKE & TriKE therapies are composed of a similar design to that of BiTEs, with the addition of an anti-CD16 domain which replaces the anti-CD3 domain to target NK cell activation instead of T-cells, in the hopes of increased efficacy via NK cell immune defense.^106–109^ Finally, novel cancer vaccine development is actively underway, utilizing newly discovered TAAs with the goal of targeting both humoral and adaptive immunity.^110^ While further studies are necessary to validate the efficacy of these newly developed therapies, new treatment modalities in immunotherapy are poised to dramatically change the treatment paradigm of PCa.

## Conclusion

The rise of immunotherapy has revolutionized the treatment of a variety of cancer types over the past decade. Currently, a growing body of literature supports the emergence of T-cell redirecting bispecific antibodies targeting PCa tumor-associated antigens as a promising treatment modality. Data from early phase clinical trials investigating BiTE therapies in patients with mCRPC have consistently demonstrated anti-tumor activity. Optimism is growing as ongoing clinical trials investigating next-generation BiTE constructs targeting novel TAAs, such as STEAP-1, report promising preliminary results with both improved safety profiles and increased efficacy. The future success of BiTE therapies hinges on the ongoing development of BiTE constructs that overcome the immunosuppressive tumor microenvironment, the effective targeting of novel TAAs with limited OTOT, as well as the development of strategies to mitigate immune-related toxicities, namely CRS. The final results from multiple ongoing clinical studies are eagerly awaited, though ultimately more studies will need to determine the optimal space within the PCa disease continuum where therapy will be most effective, as well as investigations into combination therapies which may show treatment synergy and improved durability.
